# Desialylation and Apoptosis in Immune Thrombocytopenia: Implications for Pathogenesis and Treatment

**DOI:** 10.3390/cimb46110709

**Published:** 2024-10-24

**Authors:** Shiying Silvia Zheng, José Sail Perdomo

**Affiliations:** 1 Haematology Research Unit, St. George and Sutherland Clinical Campuses, School of Medicine & Health, University of New South Wales, Kogarah, NSW 2217, Australia; silvia.zheng@health.nsw.gov.au; 2 Department of Haematology, St. George Hospital, Kogarah, NSW 2217, Australia; 3 Haematology Research Group, Central Clinical School, Faculty Medicine and Health, University of Sydney, Sydney, NSW 2006, Australia

**Keywords:** immune thrombocytopenia, platelets, autoantibodies, desialylation, apoptosis

## Abstract

Immune thrombocytopenia (ITP) is an autoimmune disease in which platelet autoantibodies play a significant role in its pathogenesis. Regulatory T cell dysfunction and T cell-mediated cytotoxicity also contribute to thrombocytopenia. Current therapies are directed towards immune suppression and modulation as well as stimulation of platelet production with thrombopoietin receptor agonists. Additional mechanisms of the pathogenesis of ITP have been suggested by recent experimental data. One of these processes, known as desialylation, involves antibody-induced removal of terminal sialic acid residues on platelet surface glycoproteins, leading to hepatic platelet uptake and thrombocytopenia. Apoptosis, or programmed platelet death, may also contribute to the pathogenesis of ITP. The extent of the impact of desialylation and apoptosis on ITP, the relative proportion of patients affected, and the role of antibody specificity are still the subject of investigation. This review will discuss both historical and new evidence of the influence of desialylation and apoptosis in the pathogenesis of ITP, with an emphasis on the clinical implications of these developments. Further understanding of both platelet desialylation and apoptosis might change current clinical practice and improve patient outcomes.

## 1. Introduction

Immune thrombocytopenia (ITP) is an acquired autoimmune disease characterised by enhanced platelet destruction and impaired platelet production. Patients can present with no known predisposing condition (primary ITP) or due to an associated disorder such as malignancy, infection, and autoimmune disease (secondary ITP) [1]. Drug-induced ITP is also well recognised [2]. There are three phases of the disease: newly diagnosed (<3 months), persistent (3–12 months), and chronic (>12 months) [3]. ITP affects both children and adults. Adult ITP is typically chronic. Only about 20–30% of cases achieve long-term remission, and many patients require long-term therapy. Primary ITP comprises approximately 80% of all adult ITP cases [4]. On the other hand, paediatric ITP is often triggered by viral infections and is usually acute and self-limiting, with 80% of children experiencing spontaneous remission. In children, treatment is often unnecessary [1,5]. ITP is a heterogeneous disease. Some patients require multiple lines of therapy, while others are managed by observation only. As a result, the quality of life for patients is affected to different degrees.

In addition to its clinical heterogeneity, ITP is a complex disease, and no single proposed mechanism can fully explain its pathogenesis. Early and current data suggest that autoimmunity is responsible for causing thrombocytopenia [6,7,8,9]. Over the past decades, further evidence has emerged, showing that the dysregulation of the immune system is multifactorial. In addition to the production of antiplatelet antibodies by B and plasma cells, regulatory T cell dysfunction and cytotoxic T cell-mediated platelet destruction also contribute to the pathogenesis of ITP. More recent advances further demonstrate that autoimmunity shortens platelet survival through the modification of platelet surface glycans and may hasten platelet programmed cell death.

This review will focus on the key aspects of recent research regarding platelet desialylation and apoptosis, their relevance to ITP pathology, and the diagnostic and treatment implications.

## 2. Platelet Biogenesis and the Regulation of Lifespan

Platelets are derived from megakaryocytes, which reside in the bone marrow and to a lesser extent in the blood and lungs. It is estimated that 10^11^ platelets are generated each day in healthy adults [10,11]. Thrombopoietin (TPO) is the primary growth factor that drives thrombopoiesis. TPO stimulates the differentiation of haematopoietic stem cells into megakaryocytic precursors and their proliferation and maturation [12,13]. TPO is synthesised primarily in the liver [14], but expression in kidney and bone marrow has been demonstrated [15]. TPO binds to its c-Mpl receptors on haematopoietic stem cells and platelets [14,16], which provides a mechanism for the regulation of circulating TPO levels [17]. During thrombocytosis, more platelets, and therefore more c-Mpl receptors, are available to bind and internalise TPO. This reduces the amount of free TPO, thus decreasing both megakaryopoiesis and platelet formation. In thrombocytopenic states, for example, due to myelosuppressive therapy, less TPO is internalised by platelets and more TPO is available to bind to megakaryocytes, stimulating thrombopoiesis [18]. Curiously, TPO levels are not significantly increased in ITP compared to non-immune thrombocytopenia. This could be due to the markedly increased platelet turnover rate despite the thrombocytopenic state in ITP [19].

### 2.1. Platelet Desialylation and the Regulation of Lifespan

**Figure 1:**
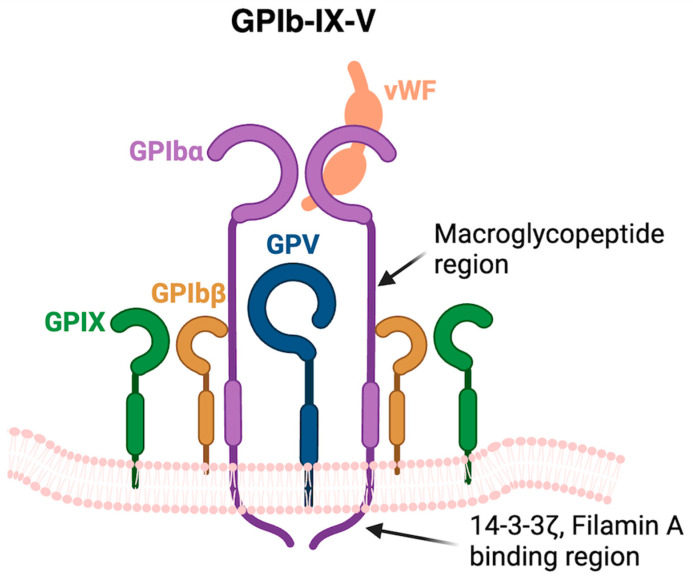
Structure of GPIb-IX-V complex. The GPIbα subunits have a heavily *O* and *N*-linked glycosylated at the N-terminus and at the mucin-like macroglycopeptide domain [24,25]. vWF, von Willebrand factor.

**Figure 2:**
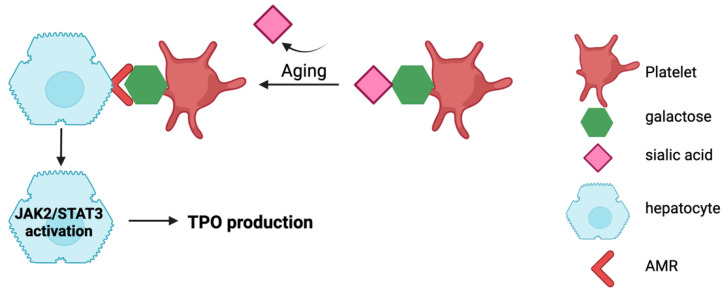
Mechanism of TPO regulation in the liver. Aged platelets lose their terminal sialic acid residues. The desialylated platelets are recognised by AMR and removed by hepatocytes, which activates the JAK2/STAT3 pathway and results in increased TPO mRNA and protein production.

Another model of TPO regulation is by controlling the TPO mRNA expression. In the last decade, a mechanism of TPO mRNA regulation was described. In a mouse model, Grozovsky and colleagues showed that aged platelets, by losing their terminal sialic acid groups attached to glycoproteins (desialylation), became recognisable by the hepatic Ashwell–Morell receptors (AMR) [20]. Glycoprotein (GP) Ibα, a highly expressed membrane-bound receptor on platelets, has an extracellular domain with an abundance of N-linked glycans (Figure 1) [21,22]. Hence, GPIbα is thought to be a major target of desialylation. The recognition of desialylated GPs by hepatocytes leads to the hepatic internalisation of platelets, which stimulates Janus kinase 2 (JAK2) and activates signal transduction and transcription 3 (STAT3). This signalling drives hepatic TPO mRNA expression (Figure 2). As a result, platelet production is increased, and platelet mass is restored [20]. In fact, a link between desialylation and platelet survival has been known for decades [23] (Table 1).

### 2.2. Platelet Apoptosis and Platelet Lifespan

**Figure 3:**
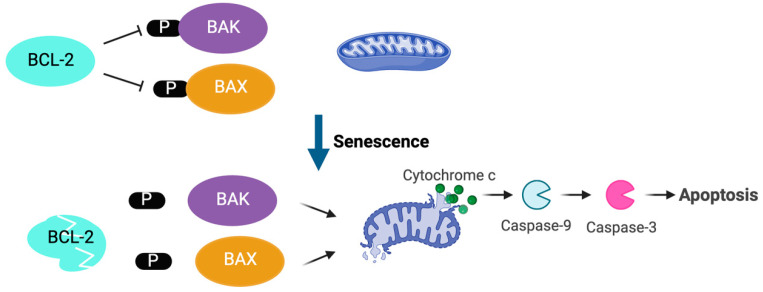
Regulation of platelet lifespan via the intrinsic apoptosis pathway. BCL2 pro-survival protein family restrains BAK and BAX activities by maintaining phosphorylation (p). Platelet senescence resulted in BCL-2 degradation, BAK and BAX dephosphorylation, which causes mitochondrial outer membrane permeabilization and initiates the apoptotic cascade.

Unlike desialylation (Table 1), the role of apoptosis was only described more recently (Table 2). In the steady state, human platelet lifespan is approximately ten days [39,40]. Intrinsic apoptosis, as triggered by non-receptor-driven events, is probably the most important regulator of platelet life span [41,42,43]. Two seminal studies showed that proteins of the BCL2 family are the key players in this pathway [42,44]. BCL-X_L_ is a pro-survival protein that sustains platelet survival by restraining the pro-death activity of the “multidomain killers” BAD, BAK1, and BAX [41]. In the cytosol, 14-3-3ζ exists in complexes with the pro-apoptotic proteins, controlling their activity by maintaining phosphorylation [45]. It has been hypothesised that, as platelets age, they gradually lose BCL-X_L_ due to degradation. However, BAK1 is degraded more slowly than BCL-X_L_, eventually reaching a point when the proapoptotic activity can no longer be restrained [42]. Free BAX transits to the mitochondria and induces mitochondrial membrane permeabilization, cytochrome c release, and activation of caspases, which results in platelet apoptosis [41] (Figure 3). Utilising a potent BCL-X_L_ antagonist ABT-737 and ^111^indium-labelled platelets whole body scintigraphy in an animal model, Zhang and colleagues identified the liver as the site of clearance of apoptotic platelets [44].

It is still unclear if platelet apoptosis and desialylation are mechanistically linked during platelet ageing. Using AMR knockout mice, a preliminary report showed that desialylated platelets are more prone to apoptosis [46]. However, the induction of platelet apoptosis did not induce platelet desialylation [46]. It is possible that the platelet ageing process involves the gradual loss of sialic acid in the circulation, followed by enhanced responsiveness to apoptotic stimuli, which result in the liberation of the pro-death proteins BAK1 and BAX and the eventual platelet apoptosis [46]. Nevertheless, stronger evidence is needed to definitively correlate desialylation with apoptosis in the regulation of platelet lifespan.

**Table 2:** Evidence for platelet apoptosis.

Comment	Reference; Year
Nucleus is not required for apoptosis	[47]; 1994
Apoptosis-like events associated with platelet activation	[48]; 1999
Anti-platelet antibodies modulate caspase activity and regulate platelet lifespan in mice	[49]; 2002
Apoptosis associated with shortened platelet survival in rabbits	[50]; 2004
Anti GPIIb antibody induces platelet apoptosis in mice	[51]; 2006
Thrombin induces platelet apoptosis	[52,53]; 2007, 2006
Apoptosis program controls platelet lifespan	[42]; 2007
Cold storage leads to platelet apoptosis	[54]; 2010
Apoptotic platelets observed in paediatric patients with ITP	[55]; 2012
Platelet apoptosis in adult ITP	[56,57]; 2018, 2016
The presence of anti-platelet antibodies predicts apoptosis in ITP	[38]; 2022

## 3. Immune Thrombocytopenia

### 3.1. Presentation

The main symptoms and signs of ITP are bleeding, ranging from minor petechiae and purpure to severe haemorrhage, such as gastrointestinal and intracranial bleeding. The severity generally correlates with the degree of thrombocytopenia and the patient’s age. Older patients with a persistently low platelet count of under 30 × 10^9^/L are at a higher risk of bleeding [58]. Notable exceptions are patients with antiplatelet autoantibodies with platelet function inhibitory effects. Occasionally, these antibodies interfere with fibrinogen binding to platelet GPIIb/IIIa or von Willebrand factor (vWF) binding to GPIb/IX receptors, resulting in severe bleeding phenotypes from acquired Glanzmann’s thrombasthenia or Bernard–Soulier syndrome, respectively [59]. These patients have severe bleeding symptoms regardless of their platelet counts. Unexpectedly, patients with ITP are also more susceptible to both venous and arterial thromboembolism [60]. In addition, patients with ITP frequently suffer from fatigue, impaired quality of life [61], and complications related to treatments [62]. Unfortunately, the mortality rates are consistently higher amongst ITP patients than in the general population [63], ranging from 1.3- to 2.2-fold [62,64,65]. Therefore, ITP is a complex disease, and understanding its pathogenesis is of importance in order to improve the current management.

### 3.2. Pathogenesis

#### 3.2.1. Fc-Dependent Pathway

It was the seminal Harrington–Hollingsworth experiment of self-infusion of ITP plasma, which led to the discovery of a humoral factor accountable for mediating platelet destruction [6,7]. Subsequently, Shulman identified that such a factor could be adsorbed by platelets and was associated with immunoglobulin G (IgG) [8]. IgM and IgA antiplatelet antibodies in the absence of IgG have been described but are rather uncommon in adult ITP [11]. In contrast, IgM may play a dominant role in acute paediatric ITP [66]. These autoantibodies are commonly directed against the platelet membrane GP complexes, and GPIIb/IIIa and GPIb/IX are most frequently targeted [67,68]. Sole specificity against GPIaIIa and GPVI antibodies is infrequent [69,70,71]. Regarding antibodies against platelet GPV, which can bind to the GPV subunit independently of the GPIb/IX complex [72], in a recent evaluation, Porcelijn reported that 5% of the 60 patients with ITP tested for anti-platelet antibodies had sole anti-GPV antibodies [73]. In fact, antibodies against more than one GP receptor are much more common in ITP [68,71].

These autoantibodies play a significant role in ITP pathogenesis. One of the most widely accepted mechanisms of platelet destruction is that opsonised platelets are phagocytosed by splenic and/or hepatic macrophages of the reticuloendothelial system via their Fc-gamma receptors (FcγR) [11,74]. Hence, this antibody-mediated platelet clearance is called the Fc-dependent pathway. Like other autoimmune diseases, ITP signifies the loss of self-tolerance. Self-reactive T cells are believed to be one of the central causes of perpetuating autoimmunity, as B cells’ ability to produce antibodies is supported by antigen-specific T cells. Studies on CD4^+^FOXP3^+^ T cells or T regulatory cells (Treg) have shown both reduction [75,76,77] and dysfunction of Treg [77] in patients with untreated ITP. Moreover, in patients with active ITP, the percentage of T-helper (Th) 17 cells is significantly higher than normal controls [78]. Interestingly, T cell clonality has been reported and implicated in treatment failure [79,80,81]. Large prospective trials are required to determine how clinicians can use this information to tailor ITP treatment strategies.

#### 3.2.2. Fc-Independent Pathway

Platelet Desialylation

Fc-independent platelet clearance has also been strongly suggested for anti-GPIbα antibodies-mediated ITP. Using monoclonal antibodies in a mouse model of ITP, Nieswandt and colleagues first demonstrated that removing the Fc fragment of anti-GPIbα monoclonal antibodies did not prevent thrombocytopenia, whereas the opposite was noted for anti-GPIIb/IIIa antibodies, denoting the Fc independence of anti-GPIbα antibodies in causing thrombocytopenia [82]. Since then, there have been further advances in the understanding of this alternative platelet clearance pathway. In 2003, Hoffmeister and colleagues observed that short-term (2 h) platelet cooling to 4 °C resulted in GPIb receptor clustering and rapid clearance of platelets by Mac-1 receptor-expressing Kupffer cells in the liver [83,84]. From 2009 to 2012, the same group discovered that the sialidase neuraminidase-1 (NEU1) was translocated to the platelet surface following longer-term (≥48 h) refrigeration of platelets. NEU1 mediates removal of terminal sialic acid residues on GPIbα, leading to platelet clearance by hepatocytes via the Ashwell–Morell receptors (AMR) [29,30,85].

More recently, Li and colleagues reported the same FcγR-independent model of platelet clearance by anti-GPIbα antibodies. Using both monoclonal antibodies and plasma from patients with ITP, they reported platelet activation secondary to GPIbα antibodies. This led to NEU1 translocation to platelet surface and platelet desialylation, as well as the resultant platelet removal by AMR on hepatocytes and hence, thrombocytopenia. This finding indicated that detection of platelet desialylation in patients with ITP might have potential diagnostic and therapeutic implications. In vivo evidence suggested that sialidase inhibitors may attenuate thrombocytopenia in ITP secondary to anti-GPIbα antibodies, while splenectomy, which reduces Fc-dependent phagocytosis of opsonised platelets, may be of little benefit for these patients [32]. However, this animal model using monoclonal antibodies may not be directly relevant to human ITP due to the lack of platelet surface FcγRIIa on murine platelets [86] and the polyclonal nature of autoantibodies from primary ITP [87]. In fact, when Cantoni et al. examined the antibody specificities and the location of platelet clearance using indium-111-labelled platelets of 93 patients with ITP, no association between antibody specificities and the site of platelet sequestration was found [88]. These observations are tempered by the fact that only four patients had sole GPIb/IX antibodies. Of those, two patients had splenic uptake while the other two had hepatic and mixed platelet sequestration patterns, respectively [88]. In a later study, Amini and colleagues examined 53 patients and reported an association between the presence of anti-GPIbα and hepatic sequestration only in patients with a platelet count under 50 × 10^9^/L [89]. Again, there were only four patients with anti-GPIbα antibodies without concomitant GPIIb/IIIa antibodies. In fact, when GPV antibodies were excluded, there was only one patient with sole anti-GPIbα antibodies [89].

Whether anti-GPIbα antibodies induce platelet desialylation and hepatic sequestration might be dose-dependent. In a murine model, Morodomi and co-workers reported that high-dose intravenous administration of monoclonal anti-mouse GPIbα antibodies led to platelet clearance by both the spleen and liver, while low-dose subcutaneous administration only led to splenic uptake [90]. Furthermore, the discrepancy between Li’s and Cantoni’s findings may also be because the true extent to which desialylation was involved in ITP pathology was unknown at the time. Recent research demonstrated that antibodies against GPIIb/IIIa are also capable of inducing platelet and megakaryocyte desialylation [35,36,37,38]. Unlike previous reports linking antibody-mediated platelet activation to desialylation [32], these latest findings did not find evidence of platelet activation [36,37,38]. Instead, when FcγR is inhibited by monoclonal antibody IV.3, desialylation is reduced [36,37]. Hence, anti-GPIIb/IIIa antibodies potentially signal through FcγRIIa to drive neuraminidase surface translocation and platelet desialylation. More importantly, these studies also confirmed the effectiveness of neuraminidase inhibitor oseltamivir in reducing platelet destruction in vivo in the setting of anti-GPIIb/IIIa antibodies-mediated ITP [37,38], providing further evidence that both anti-GPIb/IX and anti-GPIIb/IIIa antibodies may lead to platelet desialylation.

Platelet apoptosis

Platelet apoptosis has also been implicated in the pathogenic processes of ITP. In 2006, Leytin and colleagues reported that anti-GPIIb antibody injection in mice induced thrombocytopenia, platelet caspase-3 activation, enhanced phosphatidylserine (PS) exposure and mitochondrial inner transmembrane potential (ΔΨm) depolarisation. Capsase-3, PS exposure and ΔΨm depolarisation are markers of platelet apoptosis [51]. Leytin also demonstrated that treatment with IVIg prior to antibody injection ameliorated the degree of thrombocytopenia, reduced caspase-3 activation and PS exposure but not ΔΨm depolarisation. In 2012, Winkler and colleagues reported similar findings in paediatric patients with ITP [55]. However, the number of patients was small (20) and no correlation with antibody specificities was made.

More recently, Qiao and co-workers showed in vitro data of reduced expression of BCL-X_L_ and increased BAX in platelets treated with ITP plasma [91]. The researchers employed Western blot and quantitative PCR techniques to evaluate mRNA extracted from washed donor platelets treated from patients with ITP and control plasma. The results indicated a lower mRNA and protein expression of BCL-X_L_ but an increased mRNA and protein expression of BAX in platelets treated with plasma from patients with ITP when compared to control plasma [91]. However, as platelets lack nuclei, the authors did not describe the mechanism of the changes in RNA level. Possible explanations include alterations in RNA splicing of immature RNAs [92] and modification of mRNA degradation rates [93]. Nevertheless, the imbalanced expression of BCL-X_L_ and BAX at the protein level provided additional evidence of the loss of proapoptotic restrain in ITP. Again, antibody specificity was not examined in this study.

In addition to desialylation, platelet cold storage also leads to platelet apoptosis upon rewarming [54]. Like desialylation, cold storage induced platelet apoptosis may be a GPIbα driven event. Upon rewarming, the GPIbα receptor clusters, which leads to phosphorylation of its cytoplasmic domains. These phosphorylated sites have strong affinity for the above-mentioned adaptor protein 14-3-3ζ [94]. Consequently, BAX is released from 14-3-3ζ, dephosphorylated and becomes active, which leads to platelet apoptosis [54,95]. Other events, including binding of ligands such as vWF [96] and thrombin stimulation [52,53] may also result in platelet apoptosis [97].

Furthermore, both desialylated and apoptotic platelets are removed by the liver [32,44,85]. To date, a mechanistic link between platelet desialylation and apoptosis is still not fully explored (Table 1 and Table 2). From the ITP perspective, anti-GPIbα antibodies were reported to cause platelet desialylation like cold storage [32]. Yet, Leytin et al. found that monoclonal anti-GPIIb antibodies caused platelet apoptosis in a murine ITP model [51]. Whether there is an association between intrinsic apoptosis and ITP caused by anti-GPIbα and/or anti-GPIIbIIIa antibodies is still unclear. Our group analysed the differential impact of these two antibody subtypes on platelet fate, focusing on platelet desialylation and apoptosis. In contrast to Li and colleagues’ report, we found a greater proportion of patients’ sera with anti-GPIIb/IIIa antibodies led to positive neuraminidase surface translocation, while a significant number of sera from patients with sole anti-GPIbα antibodies caused platelet apoptosis [38]. Although the number of patients with sole antibody positivity was small (n = 9 for anti-GPIIb/IIIa, n = 5 for anti-GPIbα), similar findings of anti-GPIIb/IIIa antibodies indicate a greater ability to induce desialylation [35] and for anti-GPIbα to induce apoptosis [57] have been described (Table 2). Therefore, not only does the presence of platelet antibodies predict platelet desialylation and apoptosis, but the actual antibody specificity may also have implications on the ultimate platelet clearance pathway. This raises the potential of ITP treatment individualisation if such findings can be confirmed in future collaborative prospective evaluations.

T cell mediated cytotoxicity

Since not all ITP plasma-induced thrombocytopenia in healthy volunteers is from the famous Harrington experiments [6], and only about 50% of ITP patients have detectable anti-platelet antibodies [98], non-antibody-mediated mechanisms of ITP should be highlighted. Specifically, autoreactive CD8^+^ cytotoxic T cells causing platelet lysis have been reported [99,100,101]. Interestingly, Zhao et al. found a negative correlation with the detectability of platelet antibodies and the presence of platelet lysis by cytotoxic T cells, which suggested that T cell-mediated cytotoxicity could be a dominant mechanism in those patients without detectable antibodies [101]. Recently, it was shown that cytotoxic CD8^+^ T cells from patients with ITP induced platelet desialylation and apoptosis [102]. This may be a result of platelet activation through T cell receptor-mediated cytotoxic granule release [103]. Moreover, CD8^+^ cytotoxic T cells may also affect platelet production in ITP by megakaryocytes by paradoxically increasing megakaryopoiesis and decreasing apoptosis [104]. Changes in the steady state of apoptosis in megakaryocytes seem to correlate with lower platelet production [105].

## 4. Therapeutic Implications

**Figure 4:**
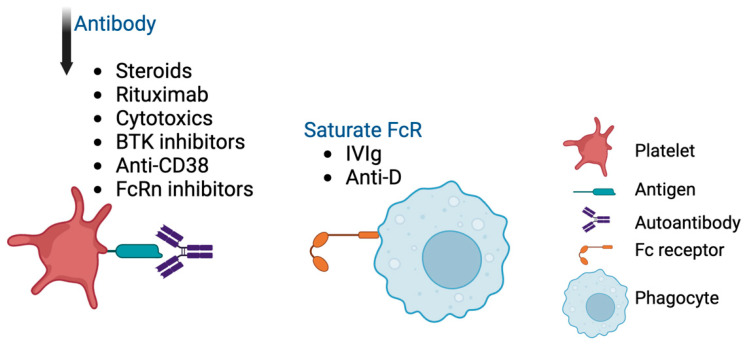
Treatments targeting the Fc-dependent pathway. BTK, Bruton kinase; FcRn, neonatal Fc receptor; IVIg, intravenous immunoglobulin.

Treatment can be divided into first-line [steroids, intravenous immunoglobulin (IVIg), and anti-Rh(D) (anti-D)], second-line [TPO receptor agonists (TPO-RA), rituximab, splenectomy] and subsequent therapies [4,106]. The currently available therapies are largely targeting the Fc-dependent pathway (Figure 4). Agents such as corticosteroids [107,108], rituximab [109,110], and cytotoxics (for example, vinca alkaloids and azathioprine [111,112]) are used to reduce antibody production. The Bruton kinase inhibitor rilzabrutinib is a promising novel anti-B cell treatment [113]. IVIg and anti-D are thought to inhibit antibody-mediated platelet clearance by saturating the FcR on macrophages [11,114]. Various combination therapies targeting this pathway have been reported [115,116]. Other novel treatments, such as anti-CD38 monoclonal antibodies to direct against plasma cells and neonatal Fc receptor inhibitors to prevent anti-platelet antibody recycling, are currently in clinical trials [117].

The dysregulation of the immune system is multifactorial, and various components of immunity are involved in perpetuating the disease. The underlying pathogenic stimuli of primary ITP often remain unknown [118]. Other mechanisms, such as megakaryocyte injury [119,120,121], reduced proplatelet formation [122,123], and complement activation [124,125,126], have implications in ITP treatment. This includes the current use of TPO RA eltrombopag [127], romiplostim [128,129], and avatrombopag [130]. Regarding complement inhibition, the Phase 1 results of a selective inhibitor sutimlimab, have recently been published: five of the 12 patients who received treatment had a durable platelet count response while on sutimlimab, which seemed to be well tolerated [131].

Oseltamivir phosphate is commonly used to treat influenza A and B with a good safety profile. It is a prodrug that, after absorption, is hydrolysed by hepatic esterases to the active form, oseltamivir carboxylate [132]. It inhibits influenza virus neuraminidase, an enzyme known to cleave the budding viral progeny from its cellular envelop attachment point (neuraminic acid) just prior to release [133]. Humans have four sialidases/neuraminidases that are homologues of viral neuraminidase, and the activity may be reduced by oseltamivir [134].

Interestingly, even before the systemic studies of ITP antibody-induced platelet desialylation, there were case reports of oseltamivir improving platelet counts in patients with ITP [33,135,136,137]. The first report, published in 2010, described a 13-year-old patient with chronic ITP who was hospitalised and treated with oseltamivir for H1N1 infection. The platelet count increased unexpectedly from 32 × 10^9^/L to over 500 × 10^9^/L [135]. In the second case published in 2015, a 69-year-old female with chronic ITP with anti-GPIb-IX antibodies was successfully managed with oseltamivir [33]. The third case described a 46-year-old female with secondary ITP from HIV infection. She was given oseltamivir for a presumed influenza infection. This was followed by normalisation of platelet count to 213 × 10^9^/L from a pre-treatment level of 9 × 10^9^/L [136]. The fourth case also reported a 51-year-old man with HIV infection on antiviral treatment and refractory ITP. His platelet count responded to combination therapy with romiplostim and oseltamivir [137]. Surprisingly, case number four also demonstrated platelet function recovery upon oseltamivir treatment using PAC1 and P-Selectin binding [137]. This is congruent with a later publication by our research group [36]. We described a patient with chronic ITP and acquired Glanzmann thrombasthenia. Not only did this patient’s antibodies inhibit platelet function, but they also led to neuraminidase translocation to the platelet surface and desialylation [36]. These findings indicate that certain anti-platelet antibodies potentially cause both platelet desialylation and platelet function inhibition.

Since the availability of more robust scientific data, systematic studies have been conducted in patients with ITP. Revilla and co-workers studied 35 patients with ITP and selected 10 of these patients with evidence of platelet desialylation for oseltamivir treatment [34]. Unlike the animal data, oseltamivir monotherapy was ineffective as none of the patients on oseltamivir-only treatment (*n* = 4) responded. However, when oseltamivir was given in combination with TPO-RA and/or immunosuppressive therapy, four out of the six patients attained a response. In contrast to the experimental data from Marini et al. [37] and our group [38], all four responders had sole anti-GPIbα antibodies [34]. No solid conclusions can be drawn from this study due to the low number of patients with single antibody specificity.

In 2021, the results of a multicentre, randomised, open-label trial using oseltamivir in combination with dexamethasone in newly diagnosed adult ITP were published [138]. The authors reported significantly improved initial response rate at day 14 in the oseltamivir group, defined as platelet count between 30 and 100 × 10^9^/L without clinically significant bleeding. The 6-month response rate was also higher in this group. However, the 12-month sustained response is not significantly different. In addition, although the duration of response was 9 months in the oseltamivir treatment group and 5 months in the control, this did not reach statistical significance. Such results may be due to the lack of power in the study. Patients with detectable anti-GPIb-IX antibodies had a poorer initial response in the dexamethasone group, but no significant difference was noted in the dexamethasone plus oseltamivir arm. The desialylation status was not examined. A sufficiently powered, well-designed, double-blind randomised clinical trial is needed to examine the potential role of oseltamivir in managing patients with newly diagnosed and refractory ITP. Its potential role in paediatric ITP and its use as a single therapy to minimise immunosuppressive and other steroid side effects are yet to be explored in clinical trials.

## 5. Conclusions

Since the initial use of splenectomy as the only treatment for ITP in the early 20th century [139], significant progress has been made in understanding the pathogenesis of ITP in recent years. This includes advances into the intricate interplay between B and T cells, as well as in the understanding of platelet antigen and autoantibody interaction. This multifaceted aspect of ITP pathology is connected to one of the most significant challenges in caring for patients with ITP: the lack of treatment individualisation. Clearly defining the platelet clearance pathway, such as desialylation and platelet apoptosis, will have important clinical implications. Patients with different autoantibodies could potentially be directed towards specific therapies. In this regard, platelet antibody testing and measuring platelet desialylation/apoptosis levels could prove clinically helpful in the treatment of ITP. Hence, the characterisation of novel mechanisms will not only contribute to the understanding of ITP’s pathogenesis but also potentially guide the development of new diagnostic tests and targeted therapies. Continued dedication to the exploration of personalised treatment should be a priority. Specifically, collaborative, high-powered studies are required to investigate treatments using neuraminidase and/or apoptosis inhibitors in randomised prospective, multicentre ITP clinical trials.

## Figures and Tables

**Table 1:** Emergence of desialylation in platelet biology.

Comment	Reference; Year
Sialic acid is present on platelets; can be cleaved with neuraminidase	[26]; 1964
Desialylated rabbit platelets are rapidly cleared in vivo; no significant changes to platelet function in vitro	[23]; 1975
Injection of desialylated platelets induced platelet production. Authors suggested that thrombopoiesis may be regulated by uptake of desialylated platelets	[27]; 1980
Sialic acid removal shortens platelet lifespan in primates	[28]; 1993
Platelets lacking sialic acid are recognised by asialoglycoprotein receptors	[29]; 2009
Cold storage leads to platelet desialylation	[30]; 2012
Platelet desialylation by anti GPIb/IX antibody	[31]; 2014
Hepatic Ashwell–Morell receptor binds and removes desialylated platelets	[20]; 2015
Anti GPIbα, not anti GPIIb/IIIa antibodies, induced desialylation and hepatic platelet uptake in mice	[32]; 2015
ITP patient with anti GPIb/IX antibodies successfully treated with oseltamivir	[33]; 2015
TPO-RAs in combination with oseltamivir induced sustained platelet production in patients with anti GPIb antibodies	[34]; 2019
Plasma from patients with ITP affected the sialylation pattern of control platelets	[35]; 2019
Desialylation induced by anti GPIIb/IIIa antibodies and is FcγRIIa-dependent	[36,37]; 2020, 2021
Destruction of human platelets induced by anti-GPIIb/IIIa antibodies was prevented with oseltamivir in a humanised mouse model of ITP	[37,38]; 2021, 2022

## Data Availability

No new data were generated in this review.
